# Supplementary material for: Hybrid machine learning models for enhanced arrhythmia detection from ECG signals using autoencoder and convolution features
Source: PLoS One. 2025 Dec 15;20(12):e0334607. doi: 10.1371/journal.pone.0334607 (PMC12704877; doi:10.1371/journal.pone.0334607)
Supplement: Attachment — Submitted filename: Plos-Response-to-Reviewers.docx [file pone.0334607.s001.docx]

**Original Manuscript ID:** PONE-D-25-09520

**Original Article Title: “**Hybrid Machine Learning Models for Enhanced Arrhythmia Detection from ECG Signals Using Autoencoder and Convolution Features”

**To:** **PLOS** ONE Editor

**Re:** Response to reviewers

Dear Editor,

Thank you for allowing a resubmission of our manuscript, with an opportunity to address the reviewers’ comments.

We are uploading (a) our point-by-point response to the comments (below) (response to reviewers, under “Author’s Response Files*”*), (b) an updated manuscript with yellow highlighting indicating changes (as “A marked-up copy of your manuscript that highlights changes made to the original version*”*), and (c) a clean updated manuscript without highlights (“An unmarked version of your revised paper without tracked changes”*).*

Best regards,
**Dr. Prince Jain**

**Reviewer#1, Concern # 1:**

Methodological clarity is lacking, particularly in the convolution feature extraction procedure. It is unclear whether convolution layers were trained separately for each classifier or reused from one trained CNN.

**Author response**: Thank you for highlighting the concern regarding the convolution feature extraction procedure. We clarify that the convolution layers were reused from a single trained CNN. These layers were not trained separately for each classifier but were utilized as a shared feature extractor to ensure consistency and efficiency across all classifiers.

We have updated the manuscript to include this explanation in the Methodology section. This addition provides greater clarity and detail about the convolution feature extraction process, addressing the reviewer’s concern.

**Reviewer#1, Concern # 2:**

The conclusions are overstated and at times inconsistent with reported results. For example, the abstract reports the top three models CFNN, AEFNN, and AEFRF with accuracies of 99.26%, 99.19%, and 99.13%, yet Table 9 (third experiment overall ranking) indicates CFNN 99.26%, AEFNN 97.91%, and AEFRF only 89.96%

**Author response:** Thank you for identifying the inconsistency in reporting results. Upon review, we realize that the abstract unintentionally presented accuracies from Table 10. We have revised the abstract to explicitly distinguish between the datasets and corresponding results to avoid ambiguity. The updated text now aligns the results with the appropriate tables and datasets.

The abstract has been updated for greater clarity and accuracy. The revised abstract now states:“It is generally observed that the top identified hybrid model Autoencoder Features with Neural Network (AEFNN) on the MIT-BIH dataset, achieving accuracies of 97.96\% and on the ECG5000 dataset the hybrid model achieved an accuracy of 99.20\%.”

**Reviewer#1, Concern # 3:**

The statistical analysis lacks rigor. No variance measures (e.g., standard deviation), confidence intervals, or statistical significance tests are provided

**Author response:**

Thank you for highlighting the need for greater statistical rigor in the analysis. While we agree that variance measures and confidence intervals are valuable, we believe that applying statistical significance tests, such as t-tests or ANOVA, may not be entirely appropriate for this study due to the following reasons:

1. **Small Sample Size:** Our experiments involve a fixed number of test samples, making statistical significance tests less reliable, as they typically require a larger sample size to draw meaningful conclusions.
2. **Deterministic Nature of ML Models:** The performance metrics reported Auc, f1-score and Accuracy are computed deterministically for each model after training, rather than being outcomes of a stochastic process. This reduces the utility of traditional significance testing, which is designed to evaluate variability in independent samples.
3. **Relevance to Model Performance:** The primary objective of our study is to compare model performance metrics directly, which is achieved through clear reporting of accuracy and related metrics rather than statistical hypothesis testing.

**Reviewer#1, Concern # 4:**

The use of mRMR for model ranking is unconventional and poorly justified. Either a more standard ranking mechanism should be used or the authors must explain their adaptation of mRMR in detail.

**Author Response:**

Thank you for raising this important concern. We fully acknowledge that mRMR (Minimum Redundancy Maximum Relevance) is traditionally used for feature selection rather than for ranking machine learning models. However, in this study, we adapted mRMR to address the challenge of ranking models based on multiple performance metrics—specifically, AUC, F1-score and Accuracy.

Our adaptation treats the performance metrics as “features” and the models as “samples.” The aim is to select metrics that are most relevant for distinguishing high-performing models while minimizing redundancy across those metrics. This is particularly useful in multi-metric evaluations, where certain metrics may be strongly correlated (e.g., Accuracy and F1-score), and a naïve averaging approach may overweight redundant information. By applying mRMR in this context, we are able to compute a composite representation of each model's performance that emphasizes informative and independent metrics, which is then further processed using the TOPSIS method for final ranking.

To address this concern, we have made the following revisions:

1. **Detailed Explanation**: We added a comprehensive description of how mRMR was adapted for model ranking in the subsection titled *“Ranking methods used for ranking the performances.”* This includes the rationale, the step-by-step procedure, and a clear justification for using this method in place of more conventional ranking approaches.
2. **Comparative Justification**: A discussion has been added outlining the limitations of standard ranking mechanisms in handling multi-metric redundancy, and how our adaptation of mRMR addresses these challenges.

These updates ensure that our methodology is clearly justified and transparent.

**Reviewer#1, Concern # 5:**

The Data Availability Statement must be corrected to accurately reflect the use of public datasets and provide accessible links. The Data Availability Statement incorrectly claims that “all relevant data are within the manuscript,” whereas the raw data are external

**Author response:** Thank you for highlighting this concern. We acknowledge the importance of accurately reflecting the data availability in the manuscript.

We have addressed this issue by adding a new section, \section{Supplementary Materials}, where we have included accessible links to the publicly available datasets (MIT-BIH Arrhythmia Dataset and ECG5000 Dataset) and a GitHub repository. The GitHub repository will host all relevant Python code and materials upon the publication of the paper. This update ensures transparency and compliance with data availability requirements.

**Reviewer#1, Concern # 6:**

The English language and writing need revision for clarity, grammar, and professionalism. Frequent typographical and grammatical errors are present (e.g., “EGG signals” instead of “ECG signals,” missing articles, tense mismatches).

**Author Response:** We sincerely appreciate the reviewer’s feedback regarding the quality of language and writing in the manuscript. We acknowledge the presence of typographical and grammatical errors and understand the importance of clear and professional communication in scientific writing.

To address this concern, we have undertaken a comprehensive review and revision of the manuscript to enhance its clarity, grammar, and professionalism. Specifically:

1. All typographical errors, including the incorrect term “EGG signals,” have been corrected to ensure accuracy.
2. Missing articles, tense mismatches, and other grammatical inconsistencies have been carefully reviewed and rectified.
3. The manuscript was thoroughly proofread and edited by a professional English language editor to ensure linguistic precision and adherence to academic writing standards.

These revisions have significantly improved the readability and quality of the manuscript

**Reviewer#1, Recommendation:**

**1.** **Clearly describe the training procedures, especially whether feature extractors were fixed or trained per classifier.**

**Author Response:** We thank the reviewer for the suggestion. The manuscript has been updated to clearly state that the feature extractors, particularly the convolutional layers in the CNN-based feature extraction pipeline, were trained once and then fixed (i.e., reused without further retraining) across all classifiers. This ensured consistent and computationally efficient feature representation across models.

This clarification has been incorporated on Page 12, under the “Feature Extraction Procedure” section.

**2.** **Add a baseline model using raw 187 features for comparison.**

**Author response:** Thank you for your valuable feedback. We would like to clarify that a baseline model using the raw 187 features has already been included in the paper. This baseline model serves as a point of comparison for evaluating the performance of the proposed hybrid models.

We appreciate your suggestion and have reviewed the manuscript to ensure that the inclusion and results of the baseline model are clearly described and appropriately highlighted for the reader.

**3.** **Include variance estimates or significance testing.**

**Author Response:** We thank the reviewer for emphasizing the need for statistical rigor. In response, we have reported standard deviation values alongside the primary performance metrics—Accuracy, F1-score, and AUC—in Tables 3 and 4. This inclusion offers a clearer representation of performance variability across models. While t-tests and ANOVA are statistically robust for moderate to large sample sizes (typically above 30 per group), their application relies on assumptions such as normality and homogeneity of variances. In our study, due to the high-dimensional nature of the extracted features and potential inter-model dependencies, these assumptions may not be fully satisfied. Therefore, we refrained from applying such tests to avoid misleading conclusions.

4. **Justify or remove mRMR; clearly explain TOPSIS setup.**

**Author Response:** The manuscript has been updated to provide a detailed justification for the use of mRMR for model ranking, highlighting its relevance to multi-metric comparisons. Additionally, the TOPSIS setup is now thoroughly explained, including the rationale, steps involved, and its integration with mRMR for ranking.

**5. Fix the abstract result discrepancies and correct all numerical and typographical issues.**

**Author Response:** The discrepancies in the abstract have been resolved, and all numerical values have been updated to align with the results presented in the manuscript. Typographical errors have also been corrected for clarity and professionalism

**6. Revise the Data Availability Statement and ensure compliance with PLOS policies**

**Author Response:** Thank you for bringing this to our attention. We have revised the Data Availability Statement to ensure full compliance with PLOS policies.

The updated statement explicitly mentions that the datasets used in this research, the MIT-BIH Arrhythmia Dataset and the ECG5000 Dataset, are publicly available and provides the corresponding access links. Additionally, we have included a new \section{Supplementary Materials} in the manuscript, where we provide these dataset links and the link to the GitHub repository. The repository will host the Python code and other relevant materials upon the publication of the paper.

This ensures transparency and accessibility in accordance with PLOS guidelines.

**Reviewer#2, Concern # 1:**

1) Feature Dimension Discrepancy & Comparison Validity: A major concern arises from the comparison between Autoencoder Features (AEF) and Convolution Features (CF). The abstract and discussion state that AEF offers enhanced performance 'for the same number of input features' or implies a direct comparison. However, the Methods section and Figure 2 describe an autoencoder bottleneck yielding 47 features (AEF), while the described 1D CNN architecture (Figure 5) results in 3008 features (47x64 from the last layer before flattening). This is a significant discrepancy. How can a fair comparison be made between models using 47 features vs. 3008 features? The authors must clarify:
Was the CNN architecture used different from the one described/diagrammed to yield ~47 features? If so, the Methods/Figures need correction. If the feature dimensions were indeed 47 (AEF) vs. 3008 (CF), the claims comparing their performance directly (e.g., 'AEF better than CF') are potentially misleading and lack a sound basis, as the classifiers are operating on vastly different input spaces. The rationale for this specific comparison needs strong justification. This ambiguity undermines the central comparison and conclusions of the study regarding the relative merits of AEF vs. CF.

**Author Response:** Thank you for your detailed and insightful observation. You are correct that the autoencoder bottleneck yields 47 features, while the CNN architecture as described in figure 3008 features (from a 47×64 output before flattening).

The CNN model which we used for feature extraction model summary.

```
━━━━━━━━━━━━━━━━━━━━━━━━━━━━━━━━━┳━━━━━━━━━━━━━━━━━━━━━━━━┳━━━━━━━━━━━━━━━┓
┃ Layer (type)                    ┃ Output Shape           ┃       Param # ┃
┡━━━━━━━━━━━━━━━━━━━━━━━━━━━━━━━━━╇━━━━━━━━━━━━━━━━━━━━━━━━╇━━━━━━━━━━━━━━━┩
│ input_layer (InputLayer)        │ (None, 187, 1)         │             0 │
├─────────────────────────────────┼────────────────────────┼───────────────┤
│ conv1d (Conv1D)                 │ (None, 187, 32)        │           128 │
├─────────────────────────────────┼────────────────────────┼───────────────┤
│ max_pooling1d (MaxPooling1D)    │ (None, 93, 32)         │             0 │
├─────────────────────────────────┼────────────────────────┼───────────────┤
│ conv1d_1 (Conv1D)               │ (None, 93, 64)         │         6,208 │
├─────────────────────────────────┼────────────────────────┼───────────────┤
│ max_pooling1d_1 (MaxPooling1D)  │ (None, 46, 64)         │             0 │
├─────────────────────────────────┼────────────────────────┼───────────────┤
│ flatten (Flatten)               │ (None, 2944)           │             0 │
├─────────────────────────────────┼────────────────────────┼───────────────┤
│ dense (Dense)                   │ (None, 128)            │       376,960 │
├─────────────────────────────────┼────────────────────────┼───────────────┤
│ dense_1 (Dense)                 │ (None, 47)             │         6,063 │
└─────────────────────────────────┴────────────────────────┴───────────────┘
```

To address this concern, we have updated Figure 5 : “ Convoluction with Machine Learning Models for Classification.” to accurately reflect the CNN architecture used for convolutional feature extraction.

**Reviewer#2, Concern # 2** :

2) Lack of Statistical Comparison Between Models: The study ranks models based on performance metrics (Accuracy, F1, AUC) using TOPSIS and mRMR. However, it does not perform any statistical tests (e.g., McNemar's test) to determine if the observed differences in performance between the top-ranked models (e.g., CFNN 99.26% vs. AEFNN 97.91% on MIT-BIH, or AEFSVMR 99.26% vs CFNN 99.19% on ECG5000) are statistically significant. Without statistical validation, concluding that one model is definitively superior based on small numerical differences in accuracy/F1/AUC on a single test split is not robust.

**Author Response:** Thank you for this important observation. We acknowledge that statistical validation such as McNemar’s test can provide further insight into whether observed differences between classifiers are statistically significant. However, in this study, our focus was on ranking models using aggregated performance metrics (Accuracy, F1-score, and AUC) via multi-criteria decision-making techniques (mRMR and TOPSIS) to reflect overall performance trends rather than establishing strict pairwise superiority.

Although McNemar’s test is appropriate for binary classification tasks like ours, it has limitations:

- It only assesses differences in misclassification patterns and does not account for multi-metric performance.
- It assumes a sufficient number of discordant predictions between two models, which may not be the case when both models perform at near-perfect levels (e.g., >99% accuracy).
- It provides no insight into performance differences in F1-score or AUC, which are critical to our evaluation.

Therefore, while we did not perform statistical hypothesis testing like McNemar’s test, we emphasized robust ranking using multi-metric criteria to assess models from a broader and more balanced perspective.

We have clarified this methodological choice and acknowledged the limitation of not including statistical significance testing in the revised manuscript. The rationale for using mRMR and TOPSIS as multi-metric ranking methods has been expanded to better justify our comparative approach.

**Reviewer#2, Concern # 3:**

a) Insufficient Detail on Model Training and Hyperparameter Tuning:
   The manuscript lacks detail on the training process and hyperparameter selection for the ML classifiers (SVM, KNN, DT, RF, NN). Were default parameters used?
b) Was any hyperparameter optimization (e.g., grid search, random search with cross-validation) performed?
c) For instance, what K value was used for KNN?
d) What were the specific architectures and training parameters (epochs, batch size beyond AE, learning rate for NN) for the NNs (AEFNN, CFNN)?
e) What regularization or complexity controls were used for DT and RF?
f) This information is crucial for reproducibility and assessing the robustness of the reported results. The high performance could potentially be dataset-specific or dependent on specific, unreported parameter tuning.

**Author response**:

a) We thank the reviewer for pointing this out. All machine learning models used in this study — including SVM, KNN, Decision Tree (DT), Random Forest (RF), and the neural network classifiers (AEFNN, CFNN) — were initially tested using default parameters from the scikit-learn and Keras/TensorFlow libraries. This approach was chosen to maintain consistency and to benchmark base-level performance across all models.
b) Our objective is to identify the better feature not improve ML model.
c) No hyperparameter optimization (e.g., grid search or random search) was performed in this work. For the K-Nearest Neighbors (KNN) classifier, we used the default setting of k=5, which corresponds to n_neighbors=5 in scikit-learn
d) The autoencoder was trained for **1000 epochs** using a **batch size of 16** with the **Adam optimizer** (learning_rate = 0.001). We trained the autoencoder until the reconstruction loss was close to zero, and the learning dynamics were visualized in Figure 3 ("Training and Validation Loss Plot").

In case of AEFNN, CFNN we keep all parameter similar only epochs =100. Fig 9. The Loss plot of the AEFNN Model for Heart Disease Detection Using Autoencoder Features on the ECG500Dataset.

e) For **Decision Tree** and **Random Forest**, we used the default scikit-learn settings.
f) We agree with the reviewer that such information is crucial for reproducibility. As we mention before our objective is to identify the better feature not improve ML model.

**Reviewer#2, Concern # 4:**

4) Justification and Application of Ranking Methods: While TOPSIS is a recognized multi-criteria decision-making method, the rationale for choosing it specifically for this task over other methods isn't provided. More critically, the use of mRMR (Maximum Relevance Minimum Redundancy) seems unconventional for model ranking. mRMR is primarily a feature selection algorithm. The authors need to clearly explain and justify how mRMR was adapted to rank models based on performance metrics (AUC, F1, Accuracy). Its standard formulation does not directly apply to this context.

**Author response:**

Thank you for raising this important point. We appreciate the opportunity to clarify the rationale behind our selection and adaptation of the TOPSIS and mRMR ranking methods.

TOPSIS (Technique for Order of Preference by Similarity to Ideal Solution) was chosen as it is a widely accepted multi-criteria decision-making (MCDM) method that allows for ranking alternatives (in our case, ML models) based on multiple conflicting criteria (AUC, F1-score, Accuracy). It enables a balanced evaluation by identifying models that are closest to the ideal solution while being farthest from the worst-performing one. We have now added a brief justification for selecting TOPSIS over other MCDM methods (e.g., AHP, VIKOR) in the revised manuscript.

Regarding mRMR, we acknowledge that it is conventionally used for feature selection. In our study, we adapted the mRMR principle to evaluate and rank models based on their performance metrics by treating each model as a "feature" and each performance metric as a "dimension." This adaptation allowed us to assess the relevance of each model in terms of its average performance across metrics, while also minimizing redundancy among metrics that may be correlated (AUC, accuracy and F1-score). Though unconventional, this approach aims to emphasize models that offer consistently high performance across metrics without over-relying on any one metric. We have now included a detailed explanation of this adaptation in the manuscript, along with references to similar applications in recent literature.

We have updated the "Ranking Methods" subsection of the manuscript to include:

- A justification for selecting **TOPSIS** as the primary MCDM method.
- A detailed explanation of how **mRMR was adapted** for model ranking based on performance metrics.
- A clarification that this adaptation is exploratory and intended to offer a balanced perspective on model evaluation.

subsection*{Ranking methods used for ranking the performances }

**Reviewer#2, Minor Concern :**

**1.  Clarity/Language:** **The manuscript would benefit from careful proofreading to correct grammatical errors, typos, and awkward phrasing (e.g., 'instigation's more efficacious' in Discussion)."
Reference marker [?] appears on page 11, paragraph 1. This needs correction**

**Author response:**

Thank you for pointing out these issues. We have thoroughly proofread the manuscript and corrected grammatical errors, awkward phrasing, and typographical mistakes, including the example noted ("instigation's more efficacious"). Additionally, the erroneous reference marker "[?]" on page 11 has been identified and corrected. These edits improve the clarity and readability of the manuscript.

**2. Figures/Tables:** **Table 11 ('Model Comparison') is difficult to interpret. Clarify what '(lr)2-3 (lr)4-5' means and how this table represents a comparison.**

**Author response:**

Thank you for pointing out the confusion related to Table 11. The expressions \cmidrule(lr){2-3} and \cmidrule(lr){4-5} are LaTeX formatting commands used to create partial horizontal lines that visually group the columns in the rendered table. These commands were not intended to appear in the final document and have been properly compiled to display the table correctly.

**3. Clarify if the autoencoder was trained only on MIT-BIH data and then applied to extract features for both MIT-BIH and ECG5000 classification tasks, as stated on page 9, last paragraph. If so, discuss potential limitations. While standard scaling is mentioned, briefly confirm it was fitted only on the training data.**

**Author response:**

Thank you for your valuable observation. We confirm that the autoencoder was trained **only on the MIT-BIH dataset**, and the trained encoder was then used to extract features for both **MIT-BIH** and **ECG5000** classification tasks. This decision was made to explore the **generalizability** of the learned feature representations across datasets with similar data modalities (i.e., ECG signals).

We acknowledge that this approach introduces potential limitations. In particular, the performance on ECG5000 may be affected due to domain shift or distributional differences between the two datasets. We have now discussed this limitation in the manuscript, noting that while the approach demonstrates promising results, training a dataset-specific autoencoder might improve performance further.

Regarding preprocessing, we confirm that **standard scaling was fitted only on the training data** of each dataset and then applied to their respective test data. This step was carefully followed to prevent any form of data leakage

**4.  Reproducibility: Consider adding a Code Availability Statement. Sharing the code (e.g., via GitHub) would greatly enhance the reproducibility and impact of this work.**

**Author response :** Thank you for the helpful suggestion. We have added a new section titled **"Supplementary Materials"** in the manuscript, where we provide links to the datasets used and the complete source code via GitHub. This ensures full transparency and enhances the reproducibility and impact of our work.
