# Supplementary material for: Hybrid machine learning models for enhanced arrhythmia detection from ECG signals using autoencoder and convolution features
Source: PLoS One. 2025 Dec 15;20(12):e0334607. doi: 10.1371/journal.pone.0334607 (PMC12704877; doi:10.1371/journal.pone.0334607)
Supplement: Attachment — Submitted filename: Response to Reviewers Comments.docx [file pone.0334607.s002.docx]

# Revision of Manuscript:

“ Hybrid Machine Learning Models for Enhanced Arrhythmia Detection from ECG Signals Using Autoencoder and Convolution Features”

We highly appreciate the Editor, and the reviewers for their time and efforts devoted to peer reviewing of our paper “Hybrid Machine Learning Models for Enhanced Arrhythmia Detection from ECG Signals Using Autoencoder and Convolution Features ”. We have thoroughly revised the manuscript based on the comments provided by the Editor, and the reviewers of the paper. The way each comment is addressed in the paper is mentioned below point by point.

# Note on presentation of the responses:

We have prepared this detailed response document, where we reproduce the original comments received from the reviewers, and reply to each comment by explaining how the raised issues have been addressed in the revised manuscript. The changes made in the revised manuscript have been highlighted in yellow color, and the same have been reproduced in the review response file for easy reference and are presented in purple color. Also, all the references are listed at the end of the responses or whenever needed.

Following these improvements and modifications while addressing each reviewer's comment, we believe that the content and presentation of the revised manuscript have been significantly enhanced. We hope that the revision will be found satisfactory and are now looking forward to your decision.

Sincerely yours,

Subir Biswas, Prabodh Kumar Sahoo, Brajesh Kumar, Adyasha Rath, Prince

Jain, Ganpati Panda, Haipeng Liu, Xinhong Wang

# Reviewer A:

# Comment 1: Feature Comparison Clarity: The core comparison between autoencoder and convolutional features lacks balance, as the feature dimensionalities differ significantly. A fair performance comparison requires either matching the number of features or a strong justification for comparing models with such unequal input sizes

**Reply**:

Thank you for your insightful comment. We apologize for any lack of clarity regarding the feature dimensionality in our study. In our experiment, both the autoencoder-based and convolutional-based feature extraction methods were applied to the same original input size of 187 features.

To ensure a fair comparison, both methods were designed to reduce the feature space to **exactly 47 features** before being passed to the classifiers. This consistent dimensionality across both feature extraction techniques ensures that the performance comparison is balanced and not influenced by differences in input size.

We have revised the manuscript to make this point clearer in the relevant section, ensuring that readers understand that the feature sets used for classification were of equal dimensionality.

# Comment 2: Statistical Rigor: The reported accuracy and F1-scores are promising but lack statistical validation. It is critical to report standard deviations across multiple runs or conduct significance testing (e.g., McNemar’s test) to support claims of model superiority.

**Reply**: Thank you for your concern on **Statistical Rigor.** We performed the statistical validation testing McNemar’s as suggested. We added a new table “Table 12: McNemar’s Test Results Comparing Model Pairs on MIT-BIH and ECG5000 Datasets”
with a subsubsection “**Statistical Analysis”**

# Comment 3: Lack of Hyperparameter Tuning: Models were evaluated with default parameters, which may not reflect their optimal performance. Basic tuning (or cross-validation) is essential to ensure fair comparisons and meaningful conclusions.

**Reply**:

Thank you for your valuable comment. We acknowledge that hyperparameter tuning is an essential step in maximizing model performance.

In our study, we initially experimented with different parameter settings for several models. However, we observed that in many cases, altering the parameters did not lead to performance improvement and, in some instances, resulted in lower accuracy compared to the default configurations.

**The primary objective of our study is to identify the best-performing model using different feature sets across multiple machine learning and deep learning models**. Given that we evaluated 16 deep learning models, we chose to use default parameters for all models to ensure a fair and consistent comparison during the ranking process. This approach allowed us to focus on the influence of feature sets and model architectures without introducing tuning-based variability.

We have included this explanation in the revised manuscript and acknowledged it as a limitation, with a plan to incorporate more comprehensive tuning (e.g., grid search or cross-validation) in future work.

**Comment 4:** Use of mRMR for Model Ranking: The use of mRMR—traditionally a feature selection method—for model ranking is unconventional and needs clearer explanation. Please specify how it was adapted and justify its relevance in this context

**Reply:**

Thank you for your valuable comment. We agree that mRMR (Minimum Redundancy Maximum Relevance) is conventionally used for feature selection rather than for ranking machine learning models. In our study, however, mRMR is not used as the primary ranking method but rather as a supporting technique to validate and strengthen the reliability of the model rankings.

The main method used for ranking the models is **TOPSIS (Technique for Order Preference by Similarity to Ideal Solution)**, which evaluates each model based on multiple performance metrics (AUC, F1-score, and Accuracy) and provides a final ranking.

To enhance the robustness of this ranking process, we applied mRMR to assess the relevance and redundancy among the selected performance metrics. In this context, the metrics are treated as “features” and the models as “samples.” The aim is to ensure that the metrics contributing to the TOPSIS analysis are both informative and non-redundant. This step helps in verifying that the final rankings are not overly influenced by correlated metrics (e.g., Accuracy and F1-score), which could bias the results.

We have added a detailed explanation of this approach in the revised manuscript under the subsection “Ranking methods used for ranking the performances,” including the rationale, procedure, and justification for using mRMR as a complementary validation tool.

In summary, **TOPSIS is used as the primary ranking method**, while **mRMR is employed to support and validate the evaluation by identifying the most informative and independent performance metrics.**

**During this experiment, we observed an interesting finding that highlights a subtle but important point: different analytical methods can sometimes converge on similar insights, even when their underlying mechanisms are distinct.**

# Reviewer B:

**Comment 1:** The authors are commended for their thorough revisions, which have successfully ensured the fairness of the model comparisons. This is a crucial step. However, a significant concern remains regarding the statistical robustness of the final conclusions.

My understanding is that a core contribution of this paper is to "identify the three best-performing models recommended for real-time arrhythmia detection", as stated in the abstract, which contradicts the response to Reviewer #2 Concern #2: "However, in this study, our focus was on ranking models using aggregated performance metrics (Accuracy, F1-score, and AUC) via multi-criteria decision-making techniques (mRMR and TOPSIS) to reflect overall performance trends rather than establishing strict pairwise superiority." For the claim in the abstract and implied throughout the paper (the word "best" is consistently used) to be fully supported, the reported performance differences must be shown to be statistically significant.

As it stands, the ranking of models based on small numerical differences may not represent true model superiority, but rather could be an artifact of the specific train/test split used.
The authors' rationale for omitting statistical tests is noted, but it seems to overlook the key purpose of these tests in model comparison, which is to validate that one model's performance is demonstrably different from another's.
There also appears to be an inconsistency in the rebuttal, where one response argues against using variance measures, while another states that standard deviation has been added.

To substantially strengthen the manuscript and ensure its primary conclusion is fully supported by the evidence, I recommend one of the following two paths for revision:

1) Perform statistical validation.

The authors could conduct appropriate statistical tests (e.g., McNemar's test --- even with the limitations cited in the response to Reviewer #2 Concern #2 --- or paired t-tests over cross-validation folds) to scientifically validate their model rankings. This would provide strong evidence for their claims of superiority.

2) Reframe the contribution and conclusions.

Alternatively, the authors could revise their claims to better reflect the evidence. This would involve removing definitive statements about the "best" or "top" models and instead concluding that several models achieved high and statistically comparable performance. This would shift the paper's focus to an exploration of methods rather than a declaration of a definitive winner.

**Reply :**

Thank you for your significant concern on statistical analysis. We completely agree with your suggestion. We Perform McNemar’s test to scientifically validate the model ranking.

We added a new Table title “McNemar’s Test Results Comparing Model Pairs on MIT-BIH and ECG5000 Datasets”

We added a new subsubsection “Statistical Analysis” about McNemar’s test.

Also, we already amended our conclusion section to incorporate the insights from the statistical comparison
