# Supplementary material for: Hybrid machine learning models for enhanced arrhythmia detection from ECG signals using autoencoder and convolution features
Source: PLoS One. 2025 Dec 15;20(12):e0334607. doi: 10.1371/journal.pone.0334607 (PMC12704877; doi:10.1371/journal.pone.0334607)
Supplement: Attachment — Submitted filename: ECG-Reviewer-responses.docx [file pone.0334607.s003.docx]

# Revision of Manuscript:

“ Hybrid Machine Learning Models for Enhanced Arrhythmia Detection from ECG Signals Using Autoencoder and Convolution Features”

We highly appreciate the Editor, and the reviewers for their time and efforts devoted to peer reviewing of our paper “Hybrid Machine Learning Models for Enhanced Arrhythmia Detection from ECG Signals Using Autoencoder and Convolution Features ”. We have thoroughly revised the manuscript based on the comments provided by the Editor, and the reviewers of the paper. The way each comment is addressed in the paper is mentioned below point by point.

# Note on presentation of the responses:

We have prepared this detailed response document, where we reproduce the original comments received from the reviewers, and reply to each comment by explaining how the raised issues have been addressed in the revised manuscript. The changes made in the revised manuscript have been highlighted in yellow color, and the same have been reproduced in the review response file for easy reference and are presented in purple color. Also, all the references are listed at the end of the responses or whenever needed.

Following these improvements and modifications while addressing each reviewer's comment, we believe that the content and presentation of the revised manuscript have been significantly enhanced. We hope that the revision will be found satisfactory and are now looking forward to your decision.

Sincerely yours,

Subir Biswas, Prabodh Kumar Sahoo, Brajesh Kumar, Adyasha Rath, Prince

Jain, Ganpati Panda, Haipeng Liu, Xinhong Wang

**Reviewer A:** The authors have made substantial improvements in this revision, particularly by addressing fairness in feature comparisons, adding statistical validation with McNemar’s test, and clarifying the ranking methodology. The study now presents a more rigorous and reproducible framework for evaluating hybrid ML models in arrhythmia detection. Minor issues remain, including the reliance on default hyperparameters, some residual language/grammar inconsistencies, and the need to align conclusions more closely with the statistical evidence. Overall, the manuscript is much improved and makes a valuable contribution, and with minor refinements it should be suitable for publication.

**Response to Reviewer A**

We sincerely thank the reviewer for the constructive and encouraging feedback. We greatly appreciate your recognition of the substantial improvements made in this revision, particularly regarding fairness in feature comparisons, the integration of statistical validation, and the clarification of the ranking methodology. We are pleased that you find the study more rigorous and reproducible, and we carefully address the remaining minor issues below:

1. **Reliance on default hyperparameters**
   We acknowledge the importance of hyperparameter tuning in maximizing model performance. In our study, we initially experimented with different parameter settings for each model. However, we observed that altering the parameters generally did not improve performance, and in some cases even reduced accuracy compared to the default configurations. Furthermore, extensive hyperparameter tuning risked contradicting our core research objective—comparing different feature representations under a consistent and fair modeling setup. For this reason, we adopted the default configurations as a balanced approach, ensuring fairness and reproducibility across all models.
2. **Residual language/grammar inconsistencies**
   We carefully re-examined the manuscript and revised sections with residual language and grammar inconsistencies. These corrections have improved clarity, readability, and overall presentation.
3. **Alignment of conclusions with statistical evidence**
   In response to this valuable suggestion, we have expanded the Statistical Analysis section to better align conclusions with the supporting evidence. Specifically, we added two new tables (Table 12 and Table 13) reporting pairwise McNemar’s test results for the top four models on the ECG5000 and MIT-BIH datasets, respectively. In these tables, the upper triangle indicates whether differences between models are statistically significant after Holm–Bonferroni correction (α=0.05\alpha = 0.05α=0.05), while the lower triangle provides the corresponding adjusted p-values. This update ensures that our conclusions are strongly supported by statistical validation, thereby reinforcing the robustness of our findings.

We believe these refinements address the reviewer’s concerns and further strengthen the manuscript’s rigor, clarity, and reproducibility.

**Reviewer B: Review of Second Revised Manuscript**

The authors are again commended for their responsiveness and for undertaking an additional round of revisions. The commitment to addressing the reviewers' concerns is evident, particularly with the addition of a new "Statistical Analysis" section and the performance of McNemar's test as requested. This is a significant step toward strengthening the manuscript.

While these additions are welcome, the core issue regarding the statistical validation of the model rankings has not yet been fully resolved. The current implementation of the statistical analysis, while a positive step, does not address the fundamental question raised in the previous review. This feedback is intended to provide a clear, constructive, and final path to resolve this point.


The Issue with the Current Statistical Analysis

The central goal of the paper, as stated in the abstract, is to "identify the three best-performing models recommended for real-time arrhythmia detection." This implies establishing that the top-ranked models are demonstrably superior to others. The concern raised previously was that small numerical differences in accuracy (e.g., between models ranked #1, #2, #3, #4, and #5) are not sufficient evidence of superiority without statistical validation.

The newly added analysis in Table 12 compares a top-performing model (e.g., 97.96% accuracy) against a much lower-performing model (79.42% accuracy). While the test correctly finds a significant difference, this result is self-evident and does not require statistical proof. The analysis avoids the essential question: Are the top-ranked models statistically distinguishable from each other?

To make a strong claim about identifying the "best" models, demonstrating pairwise superiority among the top contenders is necessary.


A Recommended Path Forward: A Robust Statistical Comparison

To resolve this, we recommend a robust, standard procedure for comparing multiple classifiers. This will provide the necessary evidence to support the paper's main conclusions. The following steps outline a suggested methodology focused on the accuracy metric, which aligns with the reporting in the abstract and the authors' recent analysis.

Step 1: Perform Pairwise Statistical Tests with Correction
For each dataset, we suggest performing pairwise comparisons among the top five models as ranked in Table 9 and top 6 models as ranked in Table 10 (because of the natural break in accuracy between model 5 and model 6).

The rest of the methodology will be explained for the MIT data using the top 5 models. The number of tests and p-value correction will be different for the ECG-5000 data using the top 6 models.

Test: Use McNemar's test to compare the accuracy of every possible pair of models within this top-five group.

Correction: Because this involves multiple comparisons (10 tests for 5 models), a correction must be applied to the p-values to avoid an inflated risk of Type I errors. We recommend the Holm-Bonferroni method, which is a standard and powerful way to adjust for multiple tests.

Step 2: Report the Detailed Pairwise Results
The results of this analysis should be presented in a new, separate table for each dataset. This table provides the necessary transparency for the statistical claims.

Example: Below is an example of what this table might look like for the MIT-BIH dataset, using fictitious p-values for demonstration.

Table ??. Pairwise McNemar's Test for Top 5 Models on the MIT-BIH Dataset

| | AEKFNN | AEFRF | AEFSVMR | AEFSVMP |
|:----------|:------:|:-----:|:---------:|:---------:|
| AEFNN | 0.812 | 0.765 | 0.0041* | 0.0038* |
| AEKFNN | | 0.934 | 0.061 | 0.0157 |
| AEFRF | | | 0.072 | 0.063 |
| AEFSVMR | | | | 0.981 |

Caption: Results of pairwise McNemar's tests on model accuracy. Each cell contains the raw p-value. An asterisk (*) indicates a statistically significant difference at the α = 0.05 level after applying the Holm-Bonferroni correction.

Note that in this example:
0.0038 has been compared with 0.05/10=0.0050;
0.0041 has been compared with 0.05/9=0.0055;
and 0.0157 has been compared with 0.05/8=0,00625.
Since the third smallest p-value is not significant after applying the Holm-Bonferroni correction, all the comparisons with larger nominal p-values are considered insignificant.

Step 3: Summarize the Findings in a Final Statistical Rank
Finally, to make the conclusions clear, the results from the pairwise tests should be summarized by adding a new "Statistical Rank" column to the original Tables 9 and 10. This column groups models that are not statistically different from each other into performance tiers.

Example: Based on the fictitious results in the table above, AEFNN, AEKFNN, and AEFRF are statistically equivalent, so they share the same rank. AEFSVMR and AEFSVMP form the next distinct tier.

Table 9 (Modified Snippet). Overall Evaluation...

| SN | Method | ... | Rank(mRMR) | Rank(Topsis) | Statistical Rank |
|:---|:--------|:---:|:----------:|:------------:|:----------------:|
| 1 | AEFNN | ... | 1 | 1 | 1 |
| 2 | AEKFNN | ... | 2 | 3 | 1 |
| 3 | AEFRF | ... | 3 | 3 | 1 |
| 4 | AEFSVMR | ... | 4.5 | 5 | 4 |
| 5 | AEFSVMP | ... | 4.5 | 5 | 4 |

Methodological Justification

This recommended approach is well-supported in machine learning literature. For a comprehensive overview of appropriate statistical tests for comparing classifiers, we refer the authors to the following seminal papers:

Demšar, J. (2006). "Statistical Comparisons of Classifiers over Multiple Data Sets." Journal of Machine Learning Research, 7, 1-30.

Dietterich, T. G. (1998). "Approximate Statistical Tests for Comparing Supervised Classification Learning Algorithms." Neural Computation, 10(7), 1895-1923.


Conclusion

Implementing this statistical analysis will provide robust, compelling evidence for the authors' conclusions. It will definitively answer whether the proposed model rankings are numerically convenient or statistically meaningful, thereby substantially increasing the impact and scientific validity of this valuable research.

**Response to Reviewer B**

We sincerely thank you for your thoughtful and constructive feedback, and for outlining a clear methodological pathway to strengthen the statistical validation of our findings. Your detailed guidance has been invaluable in refining the rigor and reproducibility of our study.

In this revised version, we have carefully followed your Recommended Path Forward: A Robust Statistical Comparison and implemented each step as suggested:

1. **Pairwise Statistical Tests with Holm–Bonferroni Correction**
   We conducted pairwise McNemar’s tests among the top-ranked models for both datasets (top five models for the MIT-BIH dataset and top six models for the ECG-5000 dataset). To mitigate the risk of inflated Type I errors, we applied the Holm–Bonferroni correction across all pairwise comparisons.
2. **Detailed Reporting of Results**
   The results of these pairwise comparisons are presented in new tables (Tables 12 and 13). Each table clearly reports the raw p-values, with statistically significant differences (after correction) explicitly marked. This transparent reporting enables readers to assess not only the relative performance of individual models but also the robustness of the observed differences.
3. **Statistical Ranking of Models**
   To ensure that our conclusions are fully aligned with the statistical evidence, we added a new “Statistical Rank” column to the original evaluation tables. This column groups models into statistically indistinguishable tiers, thereby distinguishing between cases of true superiority and cases where models perform equivalently. This revision provides a statistically grounded justification for identifying the best-performing models, rather than relying solely on small numerical differences in accuracy.

**updated subsection “**Statistical Analysis”

To assess whether the observed differences in model performance were statistically significant, McNemar’s test was conducted on the predictions of the top-performing models for both the MIT-BIH and ECG5000 datasets.

For the MIT-BIH dataset, five models achieved accuracies above 90%. However, AEFSVMR and AEFSVMP produced identical outputs in terms of accuracy, AUC, and F1-score (as reported in Table 9). Therefore, we considered the remaining four distinct models for pairwise comparisons. Similarly, for the ECG5000 dataset, six models achieved accuracies above 98%, but two pairs (AEFSVMR vs. AEFSVMP and AEFNN vs. AEFKNN) yielded identical results (as reported in Table 10), leaving four distinct models for comparison.

Since these comparisons involve multiple hypothesis tests (six pairwise comparisons per dataset), p-values were adjusted using the Holm–Bonferroni method to control the family-wise error rate at α = 0.05. This approach provides a robust correction while retaining statistical power.

Table 13 presents the results for the MIT-BIH dataset. The upper triangle indicates whether the difference between a pair of models is statistically significant after Holm–Bonferroni correction (✓ = significant, × = not significant), while the lower triangle reports the corresponding adjusted p-values. The results show that most comparisons involving AEFNN and AEFSVMR were significant, whereas the difference between AEFKNN and AEFRF was not. This suggests that AEFNN and AEFSVMR achieved significantly stronger performance than the other models.

Table 12 summarizes the results for the ECG5000 dataset. Here, only the comparison between AEFSVMR and AEFSVML remained statistically significant after correction, indicating a meaningful difference in predictive performance between these two models. All other model pairs showed no significant differences, suggesting that their performance was statistically indistinguishable despite numerical differences in accuracy.

Overall, the statistical analysis confirms that the superior performance of certain models—specifically AEFNN and AEFSVMR on MIT-BIH, and AEFSVMR on ECG5000—is not merely due to chance. These results provide rigorous evidence that the proposed models offer genuine and statistically validated improvements over competing approaches.

By adopting this robust statistical methodology, we believe the revised manuscript now addresses the core concern you raised—that is, providing statistically meaningful validation of the top-ranked models. This strengthens the reliability of our central claim that the recommended models are not only high-performing but also demonstrably superior according to rigorous statistical testing.

We sincerely thank the reviewer for highlighting the importance of methodological justification when comparing classifiers. We have carefully reviewed the suggested seminal works by Demšar (2006) and Dietterich (1998), and we agree that these provide a strong theoretical foundation for the application of appropriate statistical tests. Following this recommendation, we have revised the methodology section to explicitly justify our chosen evaluation approach with reference to these studies. This addition strengthens the rigor and reliability of our comparative analysis.

We are grateful for your clear and constructive recommendations, which have substantially improved both the methodological rigor and the scientific impact of our work.
